# Arrest of Development

**Published:** 1870-11

**Authors:** 


### Arrest of Devolopment.

We have received the following account of arrest of development, which may interest our readers. The observer has given no theory, intimated no supposed cause, related no legend concerning such accidents; has simply given us the facts, and wisely made no comment. Unerring nature must certainly have forgotten herself in this case.

EDITOR OF THE *Buffalo Med. Journal.*

DEAR SIR,

I have the pleasure of herewith transmitting an anomalous case that occurred in my practice yesterday morning (Sunday, November 27, 1870.)

I was called to attend Mrs Hawley of this place, in her fourth confinement. Everything progressed finely, and in a short time she was delivered of a female child, without arms or legs. The body is perfectly developed. The shoulders are perfect as far as the formation is due to the clavicle and scapula. It has a normal pelvis, and on the right side over the acetabulum, is a projection about two inches long, fleshy, and terminating in a perfectly formed big toe, with nail.

The evacuations from the bowels and bladder are natural.

I saw the mother and child 10 hours after delivery, and found them doing well. The child has taken nourishment, and appears as smart as any child of its age.

The father and mother are well developed and healthy, and the other children the same.

Yours respectfully,

D. M. PRATT, M. D.

Canaseraga, N. Y.,

Nov. 27th, 1870.

